# Integrative Cross-Modal Fusion of Preoperative MRI and Histopathological Signatures for Improved Survival Prediction in Glioblastoma

**DOI:** 10.3390/bioengineering13020179

**Published:** 2026-02-03

**Authors:** Tianci Liu, Yao Zheng, Chengwei Chen, Jie Wei, Dong Huang, Yuefei Feng, Yang Liu

**Affiliations:** 1 School of Biomedical Engineering, Air Force Medical University, No. 169 Changle West Road, Xi’an 710032, China; liutianci@fmmu.edu.cn (T.L.); zhengyao0202@fmmu.edu.cn (Y.Z.); chen18631930732@163.com (C.C.); jieweiwtt@fmmu.edu.cn (J.W.); huangdong1007785@outlook.com (D.H.); fengyuefei@fmmu.edu.cn (Y.F.); 2 Innovation Research Institute, Xijing Hospital, Air Force Medical University, No. 169 Changle West Road, Xi’an 710032, China; 3 Shaanxi Provincial Key Laboratory of Bioelectromagnetic Detection and Intelligent Perception, No. 169 Changle West Road, Xi’an 710032, China

**Keywords:** glioblastoma, MRI, whole slide image, overall survival, machine learning

## Abstract

Glioblastoma (GBM) is the most common and aggressive primary brain tumor in adults, with a median overall survival of fewer than 15 months despite standard-of-care treatment. Accurate preoperative prognostication is essential for personalized treatment planning; however, existing approaches rely primarily on magnetic resonance imaging (MRI) and often overlook the rich histopathological information contained in postoperative whole-slide images (WSIs). The inherent spatiotemporal gap between preoperative MRI and postoperative WSIs substantially hinders effective multimodal integration. To address this limitation, we propose a contrastive-learning-based Imaging–Pathology Synergistic Alignment (CL-IPSA) framework that aligns MRI and WSI data within a shared embedding space, thereby establishing robust cross-modal semantic correspondences. We further construct a cross-modal mapping library that enables patients with MRI-only data to obtain proxy pathological representations via nearest-neighbor retrieval for joint survival modeling. Experiments across multiple datasets demonstrate that incorporating proxy WSI features consistently enhances prediction performance: various convolutional neural networks (CNNs) achieve an average AUC improvement of 0.08–0.10 on the validation cohort and two independent test sets, with SEResNet34 yielding the best performance (AUC = 0.836). Our approach enables non-invasive, preoperative integration of radiological and pathological semantics, substantially improving GBM survival prediction without requiring any additional invasive procedures.

## 1. Introduction

Glioblastoma is the most common primary malignant brain tumor in adults, accounting for nearly 50% of all malignant gliomas and approximately 15% of all primary malignant brain tumors [1,2]. GBM is characterized by marked intratumoral heterogeneity, a strong propensity for recurrence, and an extremely poor prognosis [3,4]. The current standard-of-care treatment for glioblastoma consists of surgical resection followed by radiotherapy and temozolomide-based chemotherapy. Nevertheless, clinical outcomes remain dismal, with a median overall survival of fewer than 15 months and a 5-year survival rate below 5% [5].

Accurate prognostic prediction in GBM patients is essential for optimizing disease management and tailoring individualized therapeutic strategies. Histopathological examination remains the gold standard for GBM diagnosis, providing not only definitive diagnostic confirmation but also rich biological insights. However, it requires invasive tissue acquisition, limiting its applicability in preoperative or longitudinal assessments. In contrast, MRI has become the cornerstone of clinical glioma evaluation owing to its non-invasive nature and superior soft-tissue contrast [6,7].

Recent advances in deep learning, particularly convolutional neural networks, have enabled end-to-end modeling for a wide range of glioma-related clinical prediction tasks using MRI data [8,9,10,11,12]. Nevertheless, most existing studies rely solely on preoperative MRI for non-invasive prognostication, thereby overlooking the critical microscopic information contained in postoperative histopathological specimens such as WSIs. The inherent temporal and spatial disconnect between preoperative MRI and postoperative WSIs, arising from the intervening surgical procedure, poses a substantial barrier to effective multimodal integration and consequently limits further gains in predictive performance.

To overcome this challenge, we propose a contrastive-learning-based framework for synergistic imaging–pathology survival prediction. Our approach establishes cross-modal semantic correspondences between paired preoperative MRI and WSIs from the same patient, thereby bridging macroscopic radiological phenotypes with microscopic histological features. Furthermore, we demonstrate the generalizability of our contrastive learning framework on a large-scale glioma dataset containing MRI-only data. This study demonstrates the feasibility of integrating imaging- and pathology-derived information at the preoperative stage without the need for additional invasive procedures and indicates that this integrative approach may improve the accuracy and robustness of survival prediction in GBM patients.

## 2. Materials and Methods

### 2.1. Data Sources and Description

This study utilized five publicly available, high-quality glioma datasets: (1) the University of Pennsylvania Health System Glioblastoma dataset (UPENN-GBM) [13]; (2) the preoperative diffuse glioma MRI dataset from the University of California, San Francisco (UCSF-PDGM) [14]; (3) the TCGA-GBM and CPTAC-GBM datasets from the 2021 Brain Tumor Segmentation (BraTS) Challenge [15,16]; (4) the longitudinal GBM dataset from Bern University Hospital, Switzerland (Lumiere) [17].

**Figure 1:**
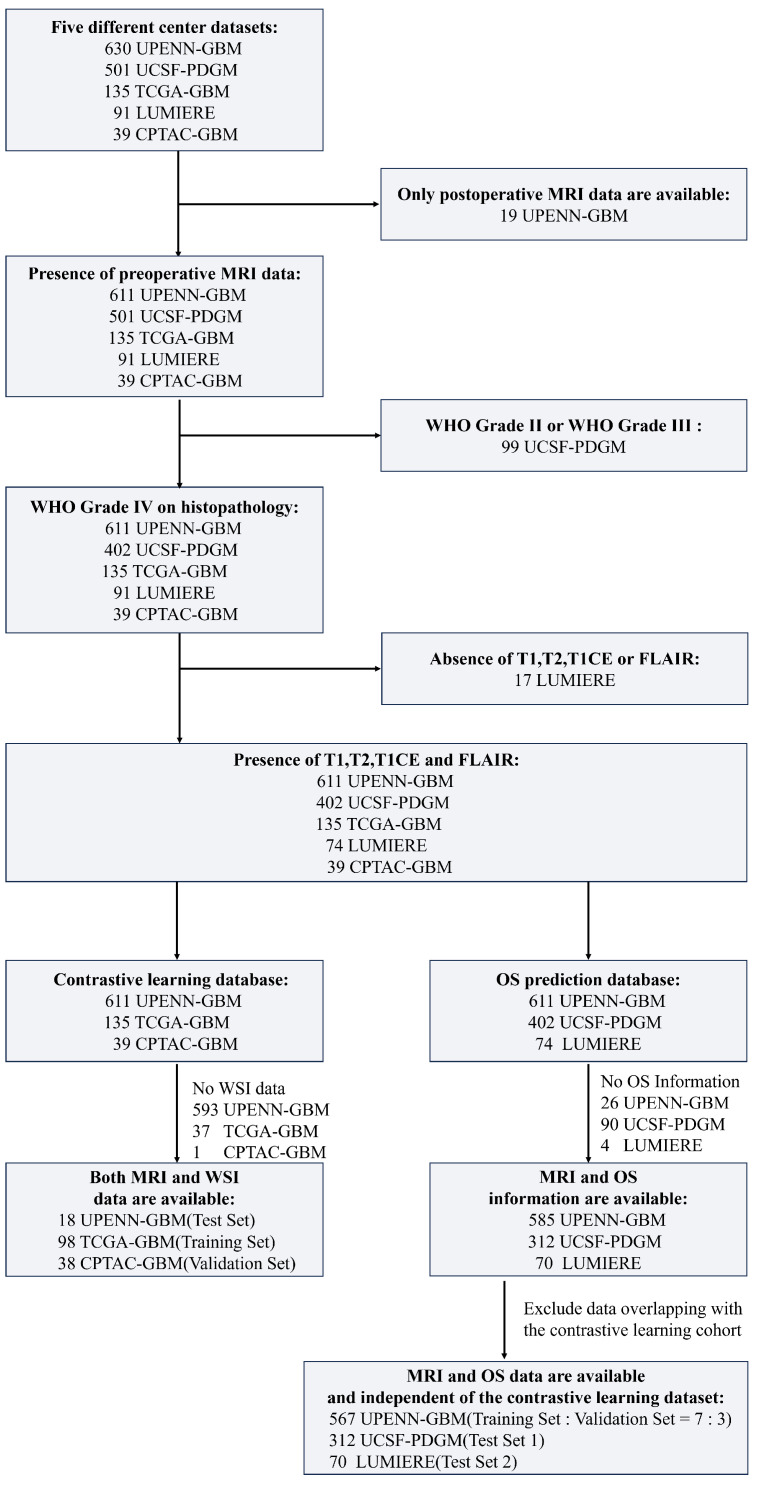
Process for inclusion of training, internal validation and two independent test sets.

The UPENN-GBM dataset contains data from 630 patients, of whom 611 have preoperative MRI and 19 have only postoperative MRI. The UCSF-PDGM dataset comprises 501 adult patients with histopathologically confirmed diffuse gliomas, including 56 WHO grade II and 43 WHO grade III cases. In this study, only patients diagnosed with WHO grade IV GBM were retained for analysis. The TCGA-GBM and CPTAC-GBM datasets—both contributing to the 2021 BraTS Challenge—include preoperative MRI scans from 135 and 39 GBM patients, respectively. The Lumiere dataset consists of longitudinal imaging data from 91 GBM patients. The detailed data inclusion process is shown in Figure 1.

In addition to MRI data, whole-slide images of histopathological specimens were obtained from The Cancer Genome Atlas (TCGA) and The Cancer Imaging Archive (TCIA) for subsets of patients in the TCGA-GBM, CPTAC-GBM, and UPENN-GBM cohorts. Specifically, the TCGA-GBM cohort includes 860 WSIs from 389 patients, the CPTAC-GBM cohort contains 527 WSIs from 200 patients, and the UPENN-GBM cohort provides 71 WSIs from 34 patients.

For MRI preprocessing, skull stripping was performed on all scans using the Brain Extraction Tool (BET) from the FMRIB Software Library (FSL 6.0.6.5) [18]. The skull-stripped T1-weighted contrast-enhanced (T1CE) images were then registered to the SRI24 atlas. Subsequently, T1-weighted (T1WI), T2-weighted (T2WI), and FLAIR sequences were co-registered to the T1CE space using the Advanced Normalization Tools (ANTs). To facilitate robust feature extraction, all images were resampled to an isotropic resolution of $1\times 1\times 1\;{\mathrm{mm}}^{3}$, resulting in a standardized volume size of 240×240×155 voxels. Intensity normalization and standardization were applied across all scans to enhance cross-scanner and multi-center comparability.

WSIs are stored in a pyramidal format. Due to variability in maximum magnification across slides (e.g., 40× vs. 20×), all WSIs were uniformly processed at the 20× magnification level. Each WSI was first partitioned into non-overlapping patches of size 256×256 pixels. Patches dominated by background regions were excluded using Otsu’s automatic thresholding method. Finally, stain normalization was applied to all retained patches using the Macenko method, which estimates and aligns the hematoxylin and eosin stain color vectors across slides. This step effectively reduces inter-institutional and inter-slide variations in staining intensity and color appearance, which are common artifacts resulting from differences in tissue processing protocols, scanner settings, and reagent batches. By minimizing these technical discrepancies, the method enhances the consistency of morphological features learned by the model and improves its generalizability across diverse clinical datasets.

### 2.2. Imaging–Pathology Collaborative Survival Prediction Framework

**Figure 2:**
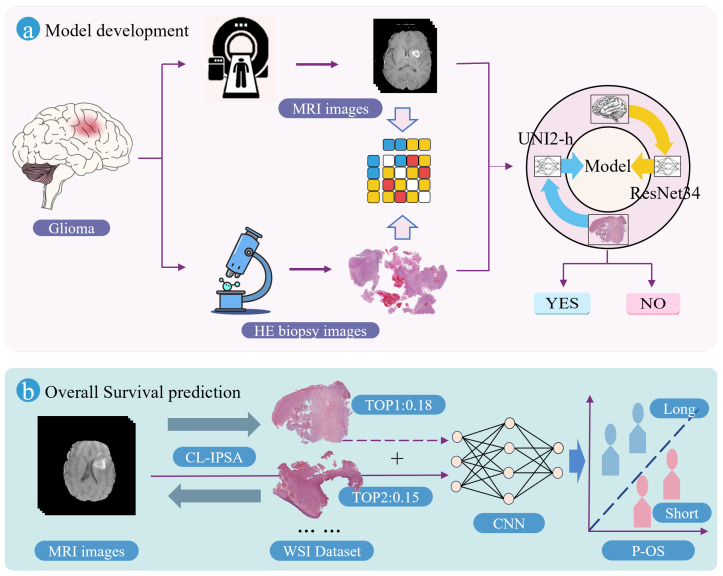
Pipeline of the Imaging–Pathology Collaborative Survival Prediction Framework. (**a**) illustrates semantic alignment of MRI and WSI features using contrastive learning; (**b**) shows preoperative MRI images matched with proxy pathology to enable accurate survival prediction using multimodal inputs.

As illustrated in Figure 2, the proposed Imaging–Pathology Collaborative Survival Prediction Framework addresses a key limitation in current research—specifically, the predominant reliance on preoperative MRI alone for survival prediction, while overlooking the rich prognostic information contained in WSIs. To bridge this gap, our framework leverages a multimodal fusion strategy to enable more accurate survival prediction for glioma patients. It consists of two core components: (1) a contrastive-learning-based imaging–pathology semantic alignment module, and (2) an overall survival prediction module. The following subsections provide detailed descriptions of these two components, along with their associated loss functions.

#### 2.2.1. Contrastive-Learning-Based Imaging–Pathology Semantic Alignment Module

**Figure 3:**
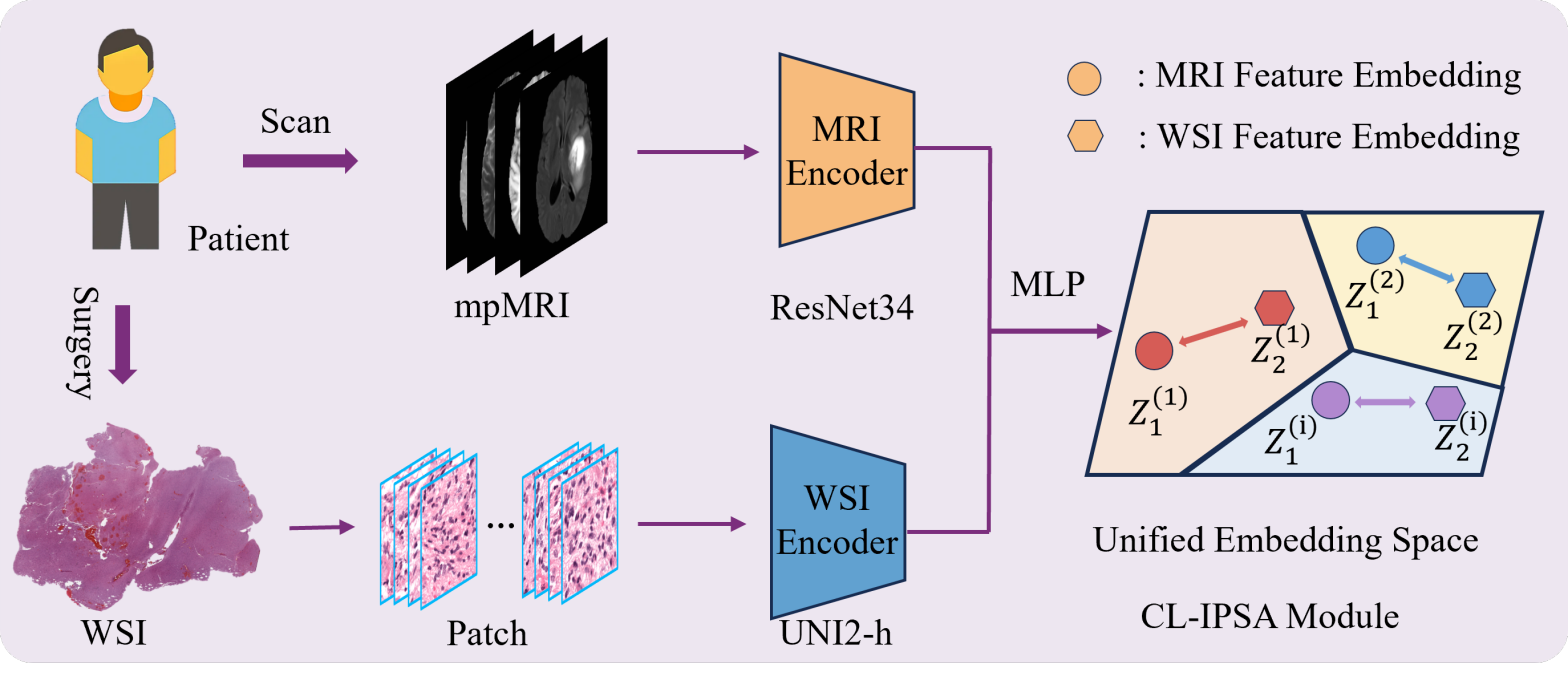
The implementation process of CL-IPSA.

The Contrastive-Learning-based Imaging–Pathology Semantic Alignment module employs two encoders, ResNet34 [19] and UNI2-h [20], to extract features from the two distinct modalities (MRI and WSI) of the same patient. Subsequently, a multi-layer perceptron (MLP) projects these heterogeneous modality-specific features into a shared embedding space, enabling cross-modal semantic alignment: it brings together multimodal feature representations from the same patient, while separating those from different patients. The module is optimized using the InfoNCE loss function to enhance both inter-modality consistency and discriminability. The schematic diagram of CL-IPSA is shown in Figure 3.

We employ different encoding architectures for MRI and WSI based on their fundamental differences in data characteristics. MRI is a three-dimensional volumetric image that requires capturing spatial structural information. The residual connections introduced by ResNet effectively alleviate the vanishing gradient problem in deep networks, enabling the model to maintain a reasonable depth while being easier to optimize and converge. Therefore, we adopt the 3D ResNet-34, which offers strong learning capacity and stability. In contrast, WSI consists of ultra-high-resolution two-dimensional pathological images, typically processed as a large collection of image patches. UNI2-h is currently the state-of-the-art model pretrained via self-supervised learning on large-scale pathological data, efficiently extracting semantically rich patch-level features and aggregating them to obtain a global representation. This tailored design fully leverages the strengths of each architecture and lays a solid foundation for subsequent cross-modal alignment and fusion.

Specifically, given a paired multimodal sample $\left(I_{\mathrm{MRI}},P_{\mathrm{WSI}}\right)$, where $I_{\mathrm{MRI}}\in R^{4\times D\times H\times W}$ denotes a 3D MRI volume comprising four sequences (T1, T1ce, T2, and FLAIR), and $P_{\mathrm{WSI}}={\left\{p_{i}\right\}}_{i=1}^{N}$ represents the set of *N* histopathology patch features pre-extracted by the UNI2-h model at 256×256 pixel resolution.

We employ two dedicated encoders to extract modality-specific semantic embeddings. A 3D ResNet34 backbone serves as the MRI encoder, whose forward pass is defined as:

(1) $$z_{\mathrm{MRI}}^{\left(512\right)}=\mathrm{GAP}\left(f_{\theta_{\mathrm{MRI}}}\left(I_{\mathrm{MRI}}\right)\right)\in R^{512}$$

where $f_{\theta_{\mathrm{MRI}}}\left(\cdot \right)$ denotes the ResNet34 feature extractor, and GAP(·) is global average pooling over spatial dimensions.

For WSI feature extraction, each patient’s WSI is represented as a variable-length patch set $P_{\mathrm{WSI}}={\left\{p_{i}\in R^{C}\right\}}_{i=1}^{N}$ with C=1536. where *C* denotes the dimensionality of each patch-level embedding. Since *N* varies across slides and patch-level embeddings exhibit substantial redundancy, an aggregation step is required to obtain a fixed-dimensional slide-level embedding.

To this end, we introduce a learnable aggregator $A_{\phi }:R^{N\times C}\to R^{C}$. Specifically, it supports a multi-head self-attention aggregation strategy.

In the MultiHeadAttentionAggregator, variable length patch sequences arising from the heterogeneous tissue content across WSIs are handled through a padding aware Transformer encoder. During batched training, input sequences are padded to the maximum length within the batch, and a binary key padding mask is generated to distinguish valid patches from padded positions. This mask is integrated into the self attention computation to suppress contributions from padding tokens, ensuring that only biologically meaningful regions influence the learned representation. A learnable [CLS] token $c\in R^{C}$ is prepended to the masked patch sequence, and the concatenated input $\left[c;p_{1},\ldots ,p_{N}\right]$ is processed by the Transformer encoder:

(2) $$\left[c;p_{1},\ldots ,p_{N}\right]\overset{\mathrm{TransformerEncoder}}{\to }\left[\tilde{c};{\tilde{p}}_{1},\ldots ,{\tilde{p}}_{N}\right],$$

where $\hat{c}$ serves as the aggregated WSI embedding. The architecture is parameterized by several key hyperparameters: the feature dimension *C* (determined by the pre-extracted patch features, we use 1536.), the number of attention heads (num_heads=4), the depth of the Transformer encoder (depth=2), the feed-forward network expansion ratio (mlp_ratio=4.0), and a dropout rate of 0.1 applied within each layer. These parameters are configured via command-line arguments during training and enable flexible modeling of spatial relationships among patches while preserving the hierarchical and non-uniform structure inherent in histopathological data.

To align the heterogeneous feature distributions and establish a shared semantic embedding space, lightweight projection heads are appended to both modalities:

(3) $$\begin{array}{cc} e_{\mathrm{MRI}} & =g_{\psi_{\mathrm{MRI}}}\left(z_{\mathrm{MRI}}^{\left(512\right)}\right), \end{array}$$

(4) $$\begin{array}{cc} e_{\mathrm{WSI}} & =g_{\psi_{\mathrm{WSI}}}\left(z_{\mathrm{WSI}}\right), \end{array}$$

where $g_{\psi }\left(\cdot \right)$ is a two-layer MLP (see ProjectionHead):

(5) $$g_{\psi }\left(x\right)=W_{2}\cdot \mathrm{ReLU}\left(\mathrm{LayerNorm}(W_{1}x)\right).$$

The hidden dimensions are configurable (default: 256 for MRI and 512 for WSI), and the output dimensionality is fixed to d=256. All embeddings are L2-normalized before similarity computation:

(6) $$\hat{e}=\frac{e}{{∥e∥}_{2}}.$$

Within this unified embedding space, we employ a symmetric InfoNCE contrastive loss to enable end-to-end optimization.

#### 2.2.2. Overall Survival Prediction Module

In clinical practice, newly enrolled patients typically have access only to multi-sequence MRI scans, whereas corresponding histopathological data are often unavailable. To leverage cross-modal semantic associations under this constraint, we propose a retrieval-based proxy pathology representation strategy. Specifically, for each new patient with MRI-only data, we first extract high-dimensional semantic features from their MRI using a 3D ResNet34 backbone, and use this embedding as a query vector to perform nearest-neighbor retrieval within the cross-modal semantic mapping library constructed via contrastive learning. Retrieval is performed using cosine similarity, and the WSI sample in the library that exhibits the highest semantic similarity to the query MRI is selected.

The retrieved WSI is then encoded using the UNI2-h model to obtain its slide-level histological embedding, which serves as a proxy pathology representation for the patient’s missing tissue data. A standard CNN architecture is used to extract MRI features, which are concatenated channel-wise with the retrieved WSI proxy embedding to form a unified multimodal representation. This joint representation is subsequently fed into a classification head to predict overall survival outcomes for glioma patients.

After training, all model parameters are frozen, and a single forward pass is performed over the entire paired dataset to construct the cross-modal embedding library:

$$K={\left\{({\hat{e}}_{\mathrm{MRI}}^{\left(k\right)},{\hat{e}}_{\mathrm{WSI}}^{\left(k\right)})\right\}}_{k=1}^{K},$$

where *K* denotes the total number of paired samples. This library preserves exact modality-wise correspondences and implicitly encodes semantic distances between non-matched samples through cosine similarity.

During inference, for a new patient with MRI-only data, the proxy pathology representation is obtained via maximum similarity matching:

(7) $${\hat{e}}_{\mathrm{WSI}}^{\mathrm{proxy}}=\arg\max_{{\hat{e}}_{\mathrm{WSI}}^{\left(k\right)}\in K}\left({\hat{e}}_{\mathrm{MRI}}^{\mathrm{new}}\cdot {\hat{e}}_{\mathrm{WSI}}^{\left(k\right)}\right)$$

This proxy representation can then be seamlessly integrated into overall survival prediction, effectively mitigating limitations imposed by the absence of real WSI data.

### 2.3. Symmetric InfoNCE Loss Function

In the context of multimodal representation learning for glioma diagnosis, where MRI and WSI data are paired at the patient level, the symmetric InfoNCE loss provides a principled framework to align embeddings across modalities. Given two batches of normalized feature embeddings $z_{1}\in R^{N\times d}$ (from MRI) and $z_{2}\in R^{N\times d}$ (from WSI), each containing *N* samples corresponding to *N* distinct patients, the symmetric InfoNCE loss is formulated as the average of two directional contrastive losses:

(8) $$L=\frac{1}{2}\left(L_{\mathrm{MRI}\to \mathrm{WSI}}+L_{\mathrm{WSI}\to \mathrm{MRI}}\right).$$

This symmetry ensures that the learning objective treats both modalities equally, preventing bias toward either MRI or WSI during training.

The cross-modal alignment is governed by a similarity matrix that captures pairwise affinities between embeddings from the two modalities within a batch. Let $z_{\mathrm{MRI}}^{\left(i\right)}\in R^{d}$ and $z_{\mathrm{WSI}}^{\left(j\right)}\in R^{d}$ denote the *d*-dimensional feature representations of the *i*-th MRI scan and the *j*-th whole-slide image, respectively, extracted by their respective encoders. Prior to computing similarity, both vectors are normalized to unit $\ell^{2}$ norm to ensure scale invariance and to make cosine similarity equivalent to the dot product. The scaled cosine similarities between all *N* MRI samples and *N* WSI samples in a batch are then arranged into an N×N matrix *S*, where each entry is defined as:

(9) $$S_{i,j}=\frac{1}{\tau }\cdot \left(\frac{z_{\mathrm{MRI}}^{\left(i\right)}}{∥z_{\mathrm{MRI}}^{\left(i\right)}{∥}_{2}}\right)\cdot \left(\frac{z_{\mathrm{WSI}}^{\left(j\right)}}{∥z_{\mathrm{WSI}}^{\left(j\right)}{∥}_{2}}\right),\;S\in R^{N\times N}$$

where τ>0 is a temperature hyperparameter that modulates the concentration of the resulting similarity distribution. A smaller value of τ sharpens the softmax probabilities over *S*, effectively increasing the penalty on hard negative pairs and encouraging the model to learn more discriminative features. Conversely, a larger τ yields a softer probability distribution, which can improve training stability—particularly when the batch size is limited or during early training stages—by providing smoother gradients. This temperature-scaled similarity matrix serves as the foundation for the subsequent bidirectional contrastive objective.

Building upon the similarity matrix *S* defined in Equation (9), we first formulate a unidirectional contrastive loss that treats MRI embeddings as query anchors. Specifically, for each patient *i* in a batch of size *N*, the model is tasked with identifying the corresponding WSI embedding—its positive match—among all *N* WSI candidates, which include one true positive (j=i) and N−1 negative samples (j≠i). This is achieved by applying a softmax function over the *i*-th row of *S*, which encodes the affinities between the *i*-th MRI anchor and every WSI in the batch. The resulting loss, denoted $L_{\mathrm{MRI}\to \mathrm{WSI}}$, is the average negative log-likelihood of correctly retrieving the positive pair:

(10) $$L_{\mathrm{MRI}\to \mathrm{WSI}}=-\frac{1}{N}\sum_{i=1}^{N}\log\left(\frac{\exp(S_{i,i})}{\sum_{j=1}^{N}\exp\left(S_{i,j}\right)}\right).$$

Minimizing this objective encourages the diagonal entries $S_{i,i}$—representing intra-patient cross-modal pairs—to dominate their respective rows in the softmax-normalized similarity distribution. Consequently, the learned representations are pulled closer for matched MRI–WSI pairs while being pushed away from mismatched inter-patient combinations, thereby enforcing modality alignment from the perspective of radiological features.

To ensure symmetric learning and prevent modality bias, we introduce a complementary contrastive loss in the reverse direction, where WSI embeddings serve as the query anchors. In this formulation, for each patient *i*, the *i*-th WSI is used to retrieve its matching MRI scan from the full set of *N* MRI embeddings in the batch. This corresponds to evaluating similarities along the *i*-th *column* of the similarity matrix *S*, since the column aggregates the affinities between all MRI samples (indexed by *j*) and the fixed WSI anchor *i*. The loss $L_{\mathrm{WSI}\to \mathrm{MRI}}$ is similarly defined as the mean negative log-probability of the correct match under a column-wise softmax:

(11) $$L_{\mathrm{WSI}\to \mathrm{MRI}}=-\frac{1}{N}\sum_{i=1}^{N}\log\left(\frac{\exp(S_{i,i})}{\sum_{j=1}^{N}\exp\left(S_{j,i}\right)}\right).$$

Note that while the numerator remains the same diagonal term $\exp(S_{i,i})$, the denominator now sums over the *i*-th column ($\sum_{j}\exp\left(S_{j,i}\right)$), reflecting the reversed query role. Optimizing this loss enforces consistency when querying from the pathological modality, ensuring that the joint embedding space is not only aligned but also bidirectionally coherent. This symmetry is crucial for robust multimodal retrieval and representation learning, especially in scenarios where either modality may be used as the input query at test time.

In both cases, the numerator corresponds to the similarity of the positive pair— embeddings from the same patient—while the denominator aggregates similarities over all possible pairs in the batch, effectively treating every non-matching cross-patient pair as a negative example. This in-batch negative sampling strategy leverages the full batch as a source of contrasting information without requiring external memory banks.

Within the similarity matrix *S*, the diagonal entries $S_{i,i}$ represent positive pairs, i.e., aligned MRI–WSI embeddings from the same glioma patient, whereas all off-diagonal entries $S_{i,j}$ with i≠j constitute negative pairs derived from different patients. This structure can be visualized as:

$$S=\left(\begin{array}{cccc} s_{11} & s_{12} & \ldots & s_{1N}\\ s_{21} & s_{22} & \ldots & s_{2N}\\ \vdots & \vdots & \ddots & \vdots \\ s_{N1} & s_{N2} & \ldots & s_{NN} \end{array}\right),$$

Mathematically, the symmetric InfoNCE loss exhibits several desirable properties. First, it is inherently symmetric with respect to the two modalities, ensuring that no implicit preference is placed on either MRI or WSI during optimization. As a contrastive objective, it explicitly maximizes the agreement between positive pairs while pushing apart negative ones, thereby encouraging the learned embeddings to capture shared semantic content across imaging domains. The temperature parameter τ provides fine-grained control over the concentration of the similarity distribution, influencing how aggressively the model distinguishes between correct and incorrect matches. Finally, by treating all other samples within a batch as negative examples, the loss function fully exploits available data without incurring additional computational overhead—making it highly suitable for scalable representation learning in multimodal medical imaging tasks such as glioma characterization.

### 2.4. Evaluation Metrics

Within the contrastive learning framework, we primarily adopt Recall@1 and Recall@5 as the core evaluation metrics. Recall@1 measures whether the highest-ranked prediction matches the ground-truth label, reflecting the model’s precision in single-choice settings. In contrast, Recall@5 evaluates whether the correct label appears among the five predictions with the highest confidence values, providing a more tolerant assessment of the model’s discriminative capability.

For the overall survival prediction task—formulated as a binary classification problem—we employ commonly used evaluation metrics, including accuracy, area under the receiver operating characteristic curve (AUROC), sensitivity, specificity, and the **$F_{1}$** score, to comprehensively assess model performance.

### 2.5. Implementation Details

We implemented our method using the PyTorch 2.5.0 framework on an NVIDIA A100 GPU with 80 GB of memory. Throughout the entire pipeline, strict separation of training and test sets was maintained to prevent any form of data leakage. Standard data augmentation strategies—including random horizontal flips, rotations, vertical flips, and random cropping—were applied during training to improve model robustness.

It should be noted that the tumor labels were obtained exclusively from publicly available datasets and were not generated by our team through either manual or automated segmentation. To better capture MRI features of the region of interest, for all MRI datasets, we centered the crop on the centroid of the solid tumor and extracted a volume of size 80×80×80 voxels.

During the contrastive learning phase, we applied L2 regularization with a weight decay coefficient of $1\times 10^{-5}$ to mitigate overfitting. The model was optimized using the Adam optimizer with an initial learning rate of $3\times 10^{-3}$. A cosine annealing learning-rate scheduler gradually reduced the learning rate from $3\times 10^{-3}$ to $1\times 10^{-6}$ over 100 epochs and the batch size was fixed at 15. We performed a grid search over τ∈{0.01,0.05,0.07,0.1,0.2} and selected the InfoNCE temperature parameter as 0.07 based on validation set performance.

For the overall survival prediction task, we adopted the same optimizer and learning-rate schedule as in the contrastive learning stage, but increased the batch size to 20 to better accommodate the classification setting.

## 3. Results

### 3.1. Patient Characteristics

This study utilized five high-quality glioma datasets comprising imaging data from more than 1000 patients. Strict inclusion and exclusion criteria were applied to ensure the integrity and validity of the experiments. In the contrastive-learning-based imaging–pathology semantic alignment module, three datasets were employed: TCGA-GBM (n=98), CPTAC-GBM (n=38), and UPENN-GBM (n=18), serving as the training, validation, and test sets, respectively. All patients in these datasets had paired MRI and whole-slide imaging data available.

For the overall survival prediction module, three distinct datasets were used: UPENN-GBM (n=567), UCSF-PDGM (n=312), and LUMIERE (n=70). The UPENN-GBM cohort was randomly divided into training and validation subsets using a 7:3 ratio, whereas UCSF-PDGM and LUMIERE were treated as two independent external test sets. Importantly, the datasets used in the survival prediction module did not overlap with those used for contrastive learning; only MRI scans and survival time labels were available for these patients. Detailed clinical characteristics are summarized in Table 1. Notably, due to data access restrictions, the CPTAC-GBM dataset provided only MRI and WSI images without accompanying clinical information. In addition, we cross-checked unique subject identifiers across all sources used for contrastive alignment and survival prediction and confirmed no overlap.

### 3.2. Imaging Pathology Semantic Alignment

We developed a CL-IPSA framework to enable the preoperative fusion of radiological and histopathological information for accurate overall survival prediction. Specifically, CL-IPSA takes as input the four standard MRI sequences—T1WI, T2WI, T1CE, and FLAIR—along with the corresponding WSI from the same patient. The framework is designed to learn cross-modal semantic correspondences between macroscopic imaging phenotypes and microscopic tissue architectures by aligning their latent representations within a shared embedding space. This modality alignment strategy mitigates the inherent heterogeneity between imaging and pathology data and enhances the model’s ability to generalize across diverse clinical cohorts.

**Figure 4:**
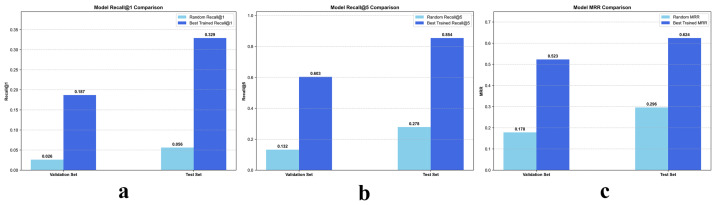
Comparison of Recall@1, Recall@5 and mean reciprocal rank between the CL-IPSA model trained with contrastive learning and the model with randomly initialized weights. (**a**–**c**) represent the detailed metrics of Recall@1, Recall@5, and Mean Reciprocal Rank before and after training across different cohorts, respectively.

As illustrated in Figure 4, the proposed contrastive learning framework, CL-IPSA, demonstrates strong performance in the semantic alignment task between MRI and whole-slide images—two intrinsically heterogeneous modalities. To assess the effectiveness of our method, we first established a baseline using a randomly initialized (untrained) version of CL-IPSA. On the validation and test sets, this random baseline achieved Recall@1 of 0.026 and 0.056, respectively, and Recall@5 of 0.132 and 0.278.

After end to end training with CL-IPSA and selecting the checkpoint with the highest validation performance, we observed substantial improvements in cross modal alignment accuracy. The trained model achieved Recall@1 scores of 0.187 and 0.329 on the validation and test sets, respectively, corresponding to approximately 7.2 fold and 5.9 fold improvements over the random baseline. Under the more permissive Recall@5 metric, the model attained scores of 0.603 on the validation set and 0.854 on the test set, representing 4.7 fold and 3.1 fold gains compared with the untrained baseline. In addition, the trained model achieved Mean Reciprocal Rank scores of 0.523 and 0.624 on the validation and test sets, respectively, which correspond to 2.9 fold and 2.1 fold improvements over the untrained model. These results demonstrate that the proposed model effectively learns semantic associations between different modalities from the same patient through contrastive learning.

**Figure 5:**
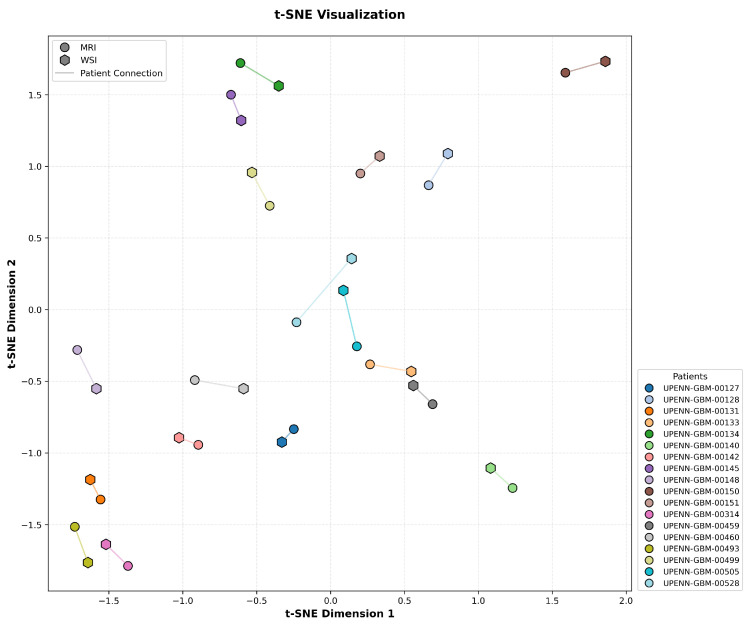
t-SNE visualization of aligned MRI and WSI embeddings from the test set.

To assess the effectiveness of cross-modal alignment in CL-IPSA, we performed a t-SNE visualization of the learned MRI and actual WSI embeddings from the UPENN-GBM test set. In Figure 5, each MRI–WSI pair from the same patient is linked by a line to indicate their shared identity. Critically, the embeddings exhibit a clear pattern consistent with the objective of contrastive learning: representations from the same patient across modalities are pulled into close proximity, while embeddings from different patients, regardless of modality, are well separated in the unified embedding space. This structure demonstrates that CL-IPSA successfully minimizes intra-patient cross-modal distance while maximizing inter-patient separation, thereby learning patient-specific, modality-invariant representations. These results provide visual confirmation that our contrastive framework achieves meaningful semantic alignment even with limited paired data, supporting its potential for clinical deployment.

The results above clearly demonstrate that CL-IPSA can effectively uncover cross-modal semantic associations between MRI and WSI, achieving deep semantic alignment through a contrastive learning mechanism without requiring explicit label supervision. This performance gain not only validates the effectiveness of the proposed contrastive loss function and modality alignment strategy but also highlights the strong generalization ability and practical applicability of our framework in multimodal medical image understanding tasks.

### 3.3. OS Time Prediction

Building upon the CL-IPSA framework, we further constructed a cross-modal semantic mapping library. For samples with MRI-only data, we performed nearest-neighbor retrieval within this library using cosine similarity to identify the WSI sample whose pathological features are most semantically similar to the given MRI embedding. The retrieved WSI is treated as a proxy pathological representation. We then systematically evaluated the improvement in overall survival (OS) prediction for GBM patients by integrating this proxy representation into several state-of-the-art CNN architectures.

As shown in Table 2, under the unimodal setting using only MRI as input, all tested CNN models demonstrated robust discriminative capability across the validation set and two independent test sets. AUC values on the validation set consistently exceeded 0.7, while those on both test sets remained above 0.6. These results indicate that, despite the inherent complexity and heterogeneity of large-scale clinical data, deep learning models can effectively extract prognostically informative features from MRI alone, enabling overall survival (OS) prediction with meaningful generalization. Specifically, SEResNet34 achieved the best performance on the validation set (AUC = 0.753), highlighting the advantage of its channel-attention mechanism in capturing informative radiological patterns. On Test Set 1, the lightweight MobileNet yielded the highest AUC (0.664), likely due to its efficient feature compression and robustness under limited-sample distributions. On Test Set 2, the multi-task learning architecture MA-MTLN achieved the best performance (AUC = 0.663), demonstrating the effectiveness of this novel design.

Furthermore, as shown in Table 3, when both the original MRI data and the proxy WSI representations derived from cross-modal semantic mapping were jointly used as model inputs, all architectures exhibited consistent and substantial performance improvements across all three evaluation sets, with average AUC gains of approximately 0.08–0.10. This finding provides strong evidence for the considerable potential of multimodal learning—integrating radiological and pathological information—for GBM survival prediction. Notably, even in the absence of real paired histopathology slides, the proxy representations generated via semantic alignment effectively complemented MRI by supplying missing microscopic tissue information, thereby enhancing the model’s understanding of tumor biology and prognostic cues. Notably, all of our reported results are based on WSI data normalized using Macenko stain normalization. When training or inference is performed without stain normalization, model performance degrades significantly. Among all compared models, SEResNet34 again demonstrated the best overall performance, achieving AUCs of 0.836, 0.736, and 0.724 on the validation set, Test Set 1, and Test Set 2, respectively—significantly outperforming all other architectures.

**Figure 6:**
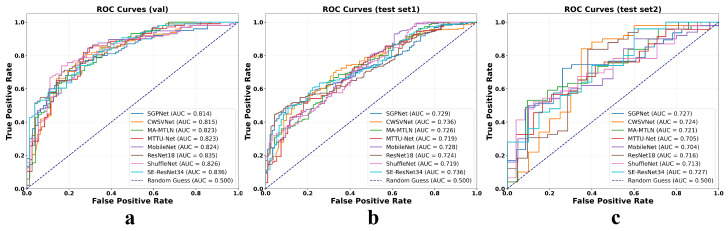
ROC curves of various CNN models using MRI and WSI as inputs: (**a**) ROC curves on the validation set, (**b**) on Test Set 1, and (**c**) on Test Set 2.

Additionally, Figure 6 displays the ROC curves for all evaluated models under both unimodal (MRI-only) and bimodal (MRI combined with proxy whole-slide image features) input conditions across three cohorts: the internal validation set and two external test sets. In each cohort, the ROC curves under the bimodal setting consistently lie above and to the left of their unimodal counterparts, indicating a clear improvement in model discriminative ability. The AUC increases uniformly across different architectures, with gains ranging from approximately 0.07 to 0.11, highlighting the added value of the cross-modal semantic mapping strategy. Notably, these performance improvements are maintained not only in the development cohort but also in geographically and technically distinct external datasets, underscoring the robustness and generalizability of the proposed multimodal fusion approach.

**Figure 7:**
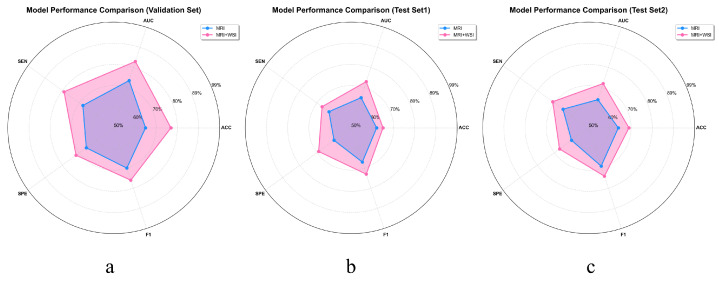
Radar charts based on comprehensive objective evaluation metrics were used to compare the performance of all models under different input modalities: (**a**) shows the performance comparison on the validation set, (**b**) on Test Set 1, and (**c**) on Test Set 2.

To more intuitively demonstrate the benefits of multimodal feature extraction over relying solely on MRI for GBM prognosis prediction, we plotted the average objective evaluation metrics across all models on the validation set and two independent test sets using radar charts. As shown in Figure 7, these charts provide a comprehensive overview of model performance and clearly highlight the substantial gains in predictive accuracy enabled by multimodal integration. This representation emphasizes the enhanced reliability and effectiveness of prognosis achieved through a multimodal strategy, rather than by assessing individual models in isolation.

### 3.4. Model Visualization

**Figure 8:**
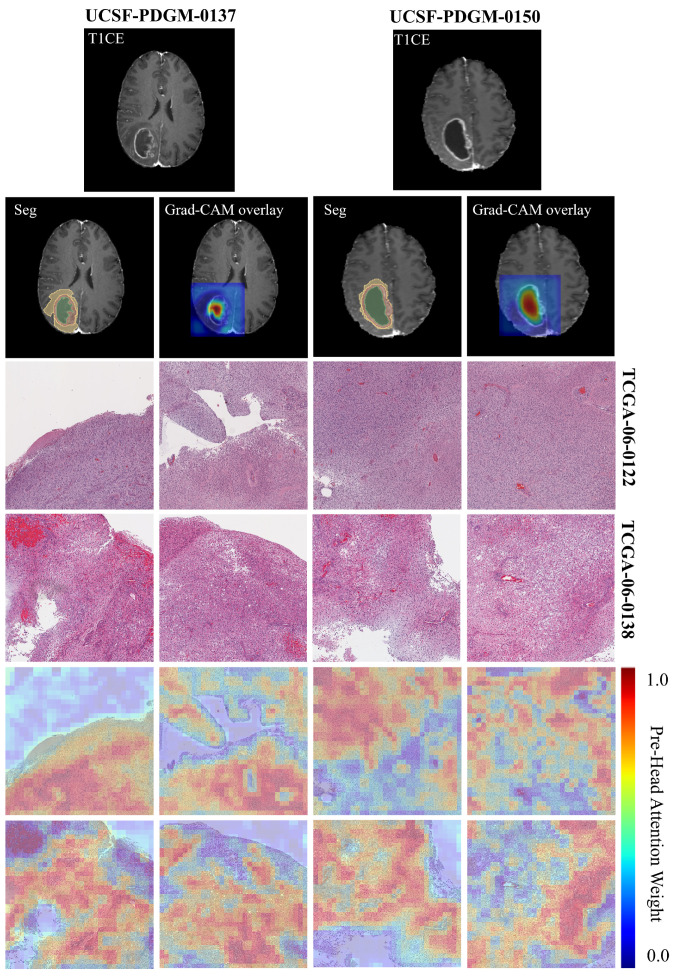
Visualization of multi-head attention maps and Grad-CAM for prognosis prediction tasks.

To better understand what histopathological features the CL-IPSA model relies on for its predictions, we perform attention visualization on H&E-stained whole-slide images. Figure 8 shows hematoxylin and eosin (H&E)-stained whole-slide images from two glioblastoma patients at 20× magnification, each divided into a 4 × 4 grid of 4096 × 4096 pixel patches. The top row displays the original histopathology, revealing hallmark features of glioblastoma such as high cellular density, nuclear atypia, microvascular proliferation, and necrotic zones. The bottom row presents multi-head attention heatmaps generated by the CL-IPSA model, where red indicates regions receiving high attention weights.

Physically, these attention patterns highlight areas with pronounced pathological abnormalities, particularly dense tumor cell clusters, pseudopalisading necrosis, and regions of abnormal vascular growth, while assigning low attention to stromal or less cellular background tissue. This selective focus reflects the model’s learned sensitivity to morphological hallmarks that are intrinsically linked to tumor aggressiveness. For example, a high nuclear-to-cytoplasmic ratio signals rapid cellular proliferation, perinecrotic palisading results from hypoxia-driven tumor cell migration, and microvascular proliferation reflects active angiogenesis. By concentrating computational resources on these biologically active zones, the attention mechanism provides a physically meaningful summary of the most prognostically relevant tissue structures within the H&E slide, enhancing both interpretability and the model’s ability to capture tumor heterogeneity directly from histology.

Furthermore, in this study, Grad-CAM was employed to visualize the regions of MRI images that contributed most strongly to the patient prognosis prediction task. The results indicate that the model predominantly attends to tumor necrotic regions. For cases UCSF-PDGM-0137 (proxy pathology TCGA-06-0122) and UCSF-PDGM-0150 (proxy pathology TCGA-06-0138), the activation maps consistently emphasize areas of necrosis. This observation suggests that imaging characteristics of necrosis, including signal heterogeneity, poorly defined margins, and atypical contrast enhancement patterns, are important predictors of adverse clinical outcomes. These findings are consistent with established pathological evidence linking tumor necrosis to aggressive tumor behavior and poor prognosis. Moreover, they support the integration of imaging features with histopathological information to achieve more interpretable and reliable prognostic models.

## 4. Discussion

The CL-IPSA framework introduced in this study enables effective synergy between imaging and pathology for GBM prognostic modeling. Its value lies not only in enhancing predictive performance but also in establishing a new paradigm for multimodal medical artificial intelligence: during training, paired multimodal data are leveraged to construct a shared cross-modal semantic space; during inference, only preoperative MRI is required, with the missing pathological modality represented via a retrieval-based proxy. This strategy effectively addresses real-world clinical constraints, such as limited access to tissue samples and delays in histopathological reporting, thereby substantially improving the practicality and deployability of the model.

Methodologically, the effectiveness of CL-IPSA arises from two key innovations. First, a symmetric InfoNCE loss function enforces bidirectional alignment between MRI and WSI embeddings, ensuring that both modalities contribute equally to the shared representation space. Second, a Transformer-based attention aggregator enables efficient and learnable patch-level feature compression for WSIs, selectively preserving the most discriminative histological regions. Notably, even with a relatively small number of paired cases in the training set, the model exhibits strong generalization across large-scale unimodal datasets—indicating that contrastive learning captures biologically meaningful patterns shared across patients and institutions, rather than overfitting to individual examples. To demonstrate the role of CL-IPSA in cross-modal semantic alignment, we compare the performance of patient prognosis prediction using WSI and MRI inputs with a trained WSI projection head against that with a randomly initialized projection head. The detailed results are provided in Table 3 and Table 4. The results indicate that WSI features processed through random projection primarily introduce noise and do not contribute to improved prognostic prediction, confirming that meaningful cross-modal integration relies on a learned, rather than random, WSI embedding.

Compared with conventional multimodal fusion approaches—such as feature concatenation, early or late fusion, and cross-attention architectures—which typically require complete multimodal inputs at inference time, the CL-IPSA framework addresses a critical clinical limitation: the frequent unavailability of pathology data when only imaging is accessible. By learning a cross-modal semantic mapping during training and retrieving reliable proxy pathology representations at test time, our method extends the benefits of multimodal modeling to a much broader patient population. Moreover, unlike recent approaches that synthesize virtual pathology images using generative models (e.g., GANs or diffusion models), our framework avoids the inherent risks of synthetic histology—including compromised structural realism, reduced cellular fidelity, and limited pathological plausibility—by directly leveraging semantic embeddings derived from real WSIs. This design substantially enhances the model’s reliability, stability, and clinical interpretability.

The proposed approach also offers several promising translational applications. First, it enables preoperative prognostic assessment that approaches the accuracy of combined imaging–pathology analysis, thereby supporting neurosurgeons in determining the extent of resection, selecting adjuvant therapies, and making clinical trial enrollment decisions. Second, for patients with recurrent GBM who are unable to undergo repeat biopsies, longitudinal MRI scans can be used to dynamically update proxy pathology features, providing a non-invasive means to monitor tumor evolution. Third, in resource-limited settings lacking neuropathology expertise, a cloud-based cross-modal library can offer proxy pathology consultations, helping to reduce disparities in diagnostic access and quality.

**Figure 9:**
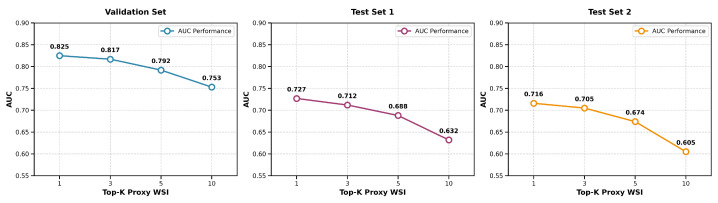
AUC performance of combining weighted top-K WSI features with MRI features for prognosis prediction across different cohorts.

To assess the robustness of proxy feature retrieval, we evaluated the impact of varying the number of nearest neighbors k in the k-NN search. We fused the top-k proxy pathological features, weighted by similarity, with MRI features to predict survival and computed the average AUC across eight models for k = 1, 3, 5, and 10. Performance peaked at k = 1 and declined steadily as k increased, indicating that additional neighbors introduce noise rather than useful information. This trend was consistent across both internal and external test sets, suggesting that the single most similar proxy WSI captures the dominant prognostic signal. The AUC values across all datasets as a function of k are summarized in Figure 9, which illustrates the performance degradation with increasing k. Thus, using k = 1 maximizes both accuracy and stability, supporting its suitability for reliable clinical application.

Nevertheless, several limitations remain. First, the quality of proxy WSI representations depends critically on the diversity and coverage of paired cases in the training cohort; rare histological subtypes absent from the training data may lead to mismatched retrievals and biased predictions. Second, the current framework does not explicitly incorporate molecular biomarkers—such as MGMT promoter methylation status or IDH mutation—that are well-established determinants of GBM prognosis. Future work could integrate genomic data to build a unified “imaging–pathology–genomics” prognostic model. Third, although stain normalization mitigates batch effects in WSI preprocessing, systematic variations arising from different tissue-processing protocols (e.g., frozen vs. paraffin sections) may still introduce domain shifts, necessitating more robust domain adaptation strategies. Fourth, this study employs a binary classification framework to predict 12-month overall survival (OS), which facilitates model evaluation but oversimplifies the continuity and complexity of patient prognosis. Future work will explore more flexible modeling strategies, such as continuous survival regression or multi-level risk stratification. Fifth, our dataset for overall survival prediction based on MRI remains limited in size. Moreover, we employed a binary classification approach rather than continuous-time survival models such as Cox regression. Addressing the class imbalance through resampling techniques may further enhance classification accuracy [28]. Sixth, during the construction of CL-IPSA, we employed the ResNet34 model. Although this network demonstrates strong learning capability and stability in processing 3D medical images, future research should compare it with other models, such as VGG and DenseNet, to identify the optimal architecture.

Another limitation of this study is that the acquisition of surrogate WSI features relies on cross-modal semantic retrieval, whose biological equivalence has not yet been systematically validated by pathology experts. Although the contrastive learning mechanism and attention visualizations indirectly support the plausibility of semantic alignment, and the observed performance gains are consistent across multiple datasets, whether the retrieved WSIs are truly similar to the query MRI in terms of key histological characteristics—such as cellular architecture, necrotic patterns, or microvascular proliferation—remains to be further evaluated by experts. Future work will incorporate blinded assessments of retrieval results by pathologists and integrate molecular pathological biomarkers to more rigorously validate the biological basis of cross-modal mapping, thereby enhancing the interpretability and clinical credibility of the model.

While we observed optimal performance with a temperature parameter of τ = 0.07 in our current experiments, this value was selected from a limited set of candidates. Given the unique characteristics of medical images, such as lower inter-class variance and domain-specific textures, the optimal temperature for contrastive learning may differ significantly from that in natural image domains. In future work, we plan to conduct a more comprehensive and systematic investigation of the temperature parameter, including broader grid searches and adaptive temperature strategies, to better tailor contrastive learning to the nuances of medical imaging data.

Although the proposed cross-modal alignment framework demonstrates significant advantages in survival prediction, the learned latent representations have not yet been systematically correlated with key molecular biomarkers, such as IDH mutation status, MGMT promoter methylation, or Ki-67 proliferation index. Therefore, it remains uncertain whether the model captures biologically meaningful signals or merely statistical associations. Future work will integrate multi-omics data to validate whether this latent space genuinely reflects the intrinsic molecular subtypes of glioblastoma, thereby advancing non-invasive imaging signatures toward interpretable, mechanism-driven tools for precision prognosis.

An emerging clinical framework that could benefit from integration with our approach is the Brain Tumor Reporting and Data System (BT-RADS) [29], which standardizes radiological reporting and risk stratification for glioma patients based on MRI features and clinical context. While our current model focuses on overall survival prediction, its learned MRI embeddings and proxy histopathological representations may capture imaging phenotypes that align with BT-RADS categories—particularly those related to tumor aggressiveness, treatment response, or progression risk. Future work should explore whether baseline multimodal embeddings derived from CL-IPSA can serve as quantitative imaging biomarkers predictive of BT-RADS classification at diagnosis or, more importantly, of BT-RADS category evolution during longitudinal follow-up. Such validation would not only enhance the clinical interpretability of our model but also support its potential use in dynamic risk assessment and standardized reporting workflows, bridging AI-driven prognostication with structured clinical decision-making systems.

Furthermore, our framework bridges MRI and WSI, it could be further enhanced by incorporating intraoperative optical techniques such as Optical Coherence Tomography (OCT) and Raman Spectroscopy. These methods provide real-time, mesoscale information on tissue microstructure and molecular composition, effectively filling the resolution gap between MRI and WSI. Integrating such data into the retrieval or fusion module during surgery could refine the proxy pathological signatures, yielding more accurate prognostic predictions and offering immediate biological feedback to guide resection. This multimodal approach would strengthen both clinical relevance and bioengineering impact.

Although the CL-IPSA framework achieved a notable improvement of 0.08 in AUC in this study, its clinical relevance should be interpreted in the context of specific decision thresholds. In the preoperative setting of glioblastoma, this improvement can translate into more accurate risk stratification. For patients near therapeutic decision boundaries, such as those of advanced age or with poor performance status, a model with an AUC of 0.75 may provide uncertain prognostic estimates, whereas an AUC of 0.83 allows more confident identification of poor prognosis. This improved discriminatory performance can support multidisciplinary clinical decision-making, helping clinicians balance aggressive surgical resection against palliative treatment strategies. Thus, the 0.08 gain in AUC reflects a meaningful improvement in the clinical utility and reliability of prognostic predictions rather than a purely numerical advance.

In summary, CL-IPSA represents not only a technical advance but also a significant step toward the vision of “non-invasive precision pathology.” Future efforts will focus on extending the framework to other solid tumors (e.g., breast or lung cancer), incorporating dynamic treatment response data, and conducting prospective clinical validation—to ultimately realize an AI-driven, personalized neuro-oncology care loop.

## 5. Conclusions

This study presents a contrastive-learning-based imaging–pathology synergistic framework for survival prediction in GBM. By establishing cross-modal semantic alignment between preoperative MRI and postoperative WSIs, the proposed method innovatively leverages a retrieval-based mechanism to generate proxy histopathological representations for patients with MRI-only data. Experimental results demonstrate that this approach significantly improves the accuracy of overall survival prediction, consistently outperforming single-modality baselines across multiple independent datasets. Crucially, the framework eliminates the need for actual tissue samples during inference, thereby offering strong clinical deployability. This work introduces a novel paradigm for multimodal medical artificial intelligence and provides a robust foundation for precision prognostication and personalized therapeutic decision-making in GBM.

## Figures and Tables

**Table 1:** Clinical Information of Patients Across Datasets.

		UPENN-GBM	UCSF-PDGM	TCGA-GBM	LUMIERE
Age		63.48±11.88	59.65±13.56	59.27±13.38	63.14±9.72
Sex	Male	350	188	74	35
	Female	235	124	43	35
OS	Less than 1 year	265	115	57	20
	More than 1 year	320	198	60	50
	Median (Months)	12.82	15.22	12.32	17.62

**Table 2:** Performance comparison of single-modal CNN models using MRI-only input on the validation set and two independent test sets.

Cohort	Metric	SGPNet [21]	CWSVNet [22]	MAMTLN [23]	MTTUNet [24]	MobileNet [25]	ResNet18 [19]	ShuffleNet [26]	SEResNet34 [27]
Validation (UPENN -GBM)	Acc.	0.687	0.690	0.688	0.693	0.707	0.678	0.667	**0.718**
	AUC	0.726	0.731	0.736	0.743	0.740	0.731	0.724	**0.753**
	Sen.	0.687	0.625	0.698	0.697	0.687	0.742	0.729	0.760
	Spec.	0.688	0.770	0.675	0.688	0.731	0.597	0.590	0.667
	F_1_	0.712	0.690	0.709	0.717	0.721	0.720	0.707	0.749
Test 1 (UCSF -PDGM)	Acc.	0.623	0.644	0.629	0.607	0.628	0.652	0.619	0.611
	AUC	0.636	0.647	0.646	0.635	**0.664**	0.657	0.643	0.653
	Sen.	0.673	0.693	0.698	0.639	0.644	0.671	0.646	0.660
	Spec.	0.528	0.548	0.505	0.546	0.595	0.614	0.566	0.518
	F_1_	0.701	0.720	0.709	0.681	0.695	0.719	0.692	0.690
Test 2 (LUMIERE)	Acc.	0.614	0.662	0.671	0.643	0.603	0.651	0.635	0.600
	AUC	0.650	0.655	**0.663**	0.632	0.632	0.622	0.626	0.651
	Sen.	0.630	0.667	0.673	0.630	0.638	0.680	0.660	0.615
	Spec.	0.583	0.636	0.667	0.667	0.500	0.538	0.562	0.556
	F_1_	0.682	0.766	0.742	0.699	0.597	0.756	0.729	0.696

**Table 3:** Performance comparison of cross-modal CNN models using MRI and WSI inputs after training projection on the validation set and two independent test sets.

Cohort	Metric	SGPNet [21]	CWSVNet [22]	MAMTLN [23]	MTTUNet [24]	MobileNet [25]	ResNet18 [19]	ShuffleNet [26]	SEResNet34 [27]
Validation (UPENN -GBM)	Acc.	0.750	0.767	0.739	0.790	0.784	0.761	0.778	0.750
	AUC	0.814	0.815	0.823	0.823	0.824	0.835	0.826	**0.836**
	Sen.	0.750	0.776	0.786	0.848	0.844	0.711	0.757	0.802
	Spec.	0.750	0.756	0.671	0.704	0.712	0.823	0.808	0.687
	F_1_	0.773	0.788	0.779	0.828	0.810	0.767	0.800	0.778
Test 1 (UCSF -PDGM)	Acc.	0.693	0.627	0.685	0.692	0.677	0.664	0.648	0.682
	AUC	0.729	0.719	0.726	0.719	0.728	0.724	0.735	**0.736**
	Sen.	0.671	0.647	0.676	0.725	0.684	0.654	0.532	0.667
	Spec.	0.723	0.547	0.710	0.621	0.670	0.672	0.904	0.691
	F_1_	0.752	0.656	0.764	0.767	0.729	0.712	0.676	0.767
Test 2 (LUMIERE)	Acc.	0.729	0.743	0.682	0.671	0.629	0.714	0.660	0.700
	AUC	0.727	0.724	0.721	0.705	0.704	0.716	0.713	**0.727**
	Sen.	0.723	0.780	0.704	0.717	0.620	0.755	0.654	0.734
	Spec.	0.739	0.650	0.619	0.583	0.650	0.619	0.667	0.600
	F_1_	0.781	0.812	0.747	0.742	0.705	0.787	0.729	0.779

**Table 4:** Performance comparison of cross-modal CNN models using MRI and WSI inputs after random projection on the validation set and two independent test sets.

Cohort	Metrics	SGPNet [21]	CWSVNet [22]	MAMTLN [23]	MTTUNet [24]	MobileNet [25]	ResNet18 [19]	ShuffleNet [26]	SEResNet34 [27]
Val	Acc.	0.665	0.614	0.670	0.659	0.705	0.642	0.653	0.648
	AUC	0.673	0.688	0.669	0.682	0.708	0.669	0.683	0.694
	Sen.	0.656	0.611	0.705	0.663	0.691	0.708	0.589	0.635
	Spe.	0.675	0.617	0.630	0.654	0.720	0.562	0.728	0.662
	F_1_	0.681	0.630	0.698	0.677	0.714	0.683	0.647	0.663
Test1	Acc.	0.553	0.601	0.559	0.585	0.603	0.524	0.591	0.518
	AUC	0.581	0.588	0.607	0.622	0.602	0.590	0.597	0.594
	Sen.	0.556	0.615	0.552	0.558	0.641	0.455	0.627	0.614
	Spe.	0.548	0.575	0.571	0.635	0.530	0.643	0.509	0.528
	F_1_	0.611	0.663	0.617	0.629	0.670	0.547	0.667	0.656
Test2	Acc.	0.600	0.586	0.614	0.629	0.642	0.614	0.614	0.643
	AUC	0.593	0.618	0.620	0.587	0.627	0.620	0.607	0.614
	Sen.	0.617	0.553	0.596	0.667	0.680	0.640	0.660	0.680
	Spe.	0.565	0.651	0.652	0.526	0.529	0.550	0.522	0.545
	F_1_	0.674	0.642	0.675	0.723	0.742	0.703	0.697	0.731

## Data Availability

All datasets are publicly available and have been cited. The UPENN-GBM, UCSF-PDGM, and TCGA-GBM datasets are from the Cancer Imaging Archive and are used with permission. The LUMIERE dataset originates from the University Hospital of Bern, Switzerland. Here are the specific download links for each dataset: UPENN-GBM: https://www.cancerimagingarchive.net/collection/upenn-gbm/ (accessed on 15 September 2025), UCSF-PDGM: https://www.cancerimagingarchive.net/collection/ucsf-pdgm/ (accessed on 15 September 2025), LUMIERE: https://springernature.figshare.com/articles/dataset/ (accessed on 15 September 2025), TCGA-GBM and CPTAC-GBM: https://www.cancerimagingarchive.net/analysis-result/rsna-asnr-miccai-brats-2021/ (accessed on 15 September 2025).

## Abbreviations

The following abbreviations are used in this manuscript:

GBM	Glioblastoma
OS	Overall Survival
WSI	Whole-Slide Image
MRI	Magnetic Resonance Imaging
CNNs	Convolutional Neural Networks
AUROC	Area Under the Receiver Operating Characteristic curve
CL-IPSA	Contrastive-Learning-based Imaging–Pathology Semantic Alignment
